# Outcomes of membranous and proliferative lupus nephritis – analysis of a single-centre cohort with more than 30 years of follow-up

**DOI:** 10.1093/rheumatology/keaa103

**Published:** 2020-04-17

**Authors:** Filipa Farinha, Ruth J Pepper, Daniel G Oliveira, Thomas McDonnell, David A Isenberg, Anisur Rahman

**Affiliations:** k1 Centre for Rheumatology, University College London; k2 Centre for Nephrology, University College London – Royal Free Campus, London, UK; k3 Internal Medicine Department, Centro Hospitalar e Universitário do Porto, Porto, Portugal

**Keywords:** systemic lupus erythematosus, lupus nephritis, outcomes, survival

## Abstract

**Objectives:**

To compare membranous lupus nephritis (MLN) and proliferative lupus nephritis (PLN) with respect to survival, demographic, clinical and laboratory characteristics; and to investigate predictors of renal and patient survival.

**Methods:**

Single-centre retrospective observational study. Patients with biopsy-proven PLN, MLN and mixed lupus nephritis were included. Groups were compared using appropriate statistical tests and survival was analysed through the Kaplan-Meier method. Cox regression analysis was performed to investigate predictors of renal and patient survival.

**Results:**

A total of 187 patients with biopsy-proven lupus nephritis (135 with PLN, 38 with MLN and 14 with mixed LN) were followed for up to 42 years (median 12 years). There was a higher proportion of MLN amongst Afro-Caribbeans than amongst Caucasians (31% *vs* 15%, *P* = 0.010). Patients with MLN had significantly lower anti-dsDNA antibodies (*P* = 0.001) and higher C3 levels (*P* = 0.018) at diagnosis. Cumulative renal survival rates at 5, 10, 15 and 20 years were 91, 81, 75 and 66% for PLN and 100, 97, 92 and 84% for MLN, respectively (*P* = 0.028). Cumulative patient survival at 5, 10, 15 and 20 years was 94, 86, 80 and 76%, with no difference between PLN and MLN. Urinary protein-creatinine ratio above 42 mg/mmol and eGFR below 76 ml/min/1.73 m^2^, one year after the diagnosis of LN, were the strongest predictors of progression to end-stage renal disease. eGFR below 77 ml/min/1.73 m^2^, at one year, development of end-stage renal disease and Afro-Caribbean ethnicity were associated with higher mortality.

**Conclusion:**

Patients with MLN and PLN differ significantly regarding serological profiles and renal survival, suggesting different pathogenesis. Renal function at year one predicts renal and patient survival.

**Rheumatology key messages**

- We compared MLN and PLN in a large multi-ethnic cohort, with a very long follow-up.
- Patients with MLN and PLN differ significantly regarding anti-dsDNA and complement levels, and renal survival.
- Renal function at year one predicts renal and patient survival in LN patients.

## Introduction

SLE is a complex and heterogeneous autoimmune systemic disease, which can affect multiple organs and systems. Lupus nephritis (LN) is one of its most severe manifestations. A recently published international inception cohort study demonstrated renal involvement in 38.3% of patients with SLE [1] but, depending on the populations studied, this proportion can be higher than 50% [2].

Despite the improvement in survival of patients with SLE and LN over the last 30 years, LN is associated with end-stage renal disease (ESRD) and death; with chronic kidney disease associated with a poorer quality of life [1].

LN is currently classified histopathologically according to the 2003 International Society of Nephrology/Renal Pathology Society (ISN/RPS) classification system [3]. More than 50% of patients have proliferative LN (PLN). Membranous LN (MLN) is less frequent, accounting for 10–20% of cases [4]. In some patients, there is a combination of membranous and proliferative changes (mixed LN) [3].

There are several studies analysing the long-term outcomes of patients with PLN [5–7], MLN [8–10], and even comparing pure PLN or pure MLN with mixed LN [11–13]. However, to our knowledge there are no recent studies directly comparing the very long-term outcomes of PLN *vs* MLN. Our objectives were to compare patients with MLN and PLN with respect to survival, demographic, clinical and laboratory characteristics; and to investigate predictors of renal and patient survival.

## Methods

### Study population

All patients with LN followed at the Rheumatology Department of University College London Hospitals (UCLH) between January 1978 and December 2017 were studied. We included not only patients diagnosed at UCLH, but also those transferred from other centres. Study subjects had to fulfil ACR 1997 [14] or SLICC 2012 [15] classification criteria for SLE, with a renal biopsy showing LN class III, IV, V or a combination of these, according to the ISN/RPS 2003 classification system [3]. We did not have any exclusion criteria. We divided the patients into three groups, according to the classification of their first renal biopsy: PLN (class III and IV), MLN (class V) and mixed LN (III+V or IV+V).

This is an observational retrospective study of medical records collected over a period of over 30 years. All data were derived from normal clinical management and no patients underwent extra questionnaires or research procedures. No individualized or identifiable data are presented in this study. Therefore, ethical approval and informed consent were not required.

### Data collection

Individual clinical files were reviewed in order to collect the following data:

Demographic data: year of birth, sex, ethnicity.

Clinical data: year of diagnosis of SLE, date of diagnosis of nephritis, number of renal biopsies, date of each renal biopsy, class of LN in each biopsy, development of ESRD, date of last visit, year and cause of death (if occurred), comorbidities, blood pressure (recorded as the proportion of visits when blood pressure was higher that the target of 130/80 mmHg), treatment with antimalarials, immunosuppressants and corticosteroids.

Laboratory data: ever-positive ANA, anti-dsDNA, anti-Sm, anti-RNP, anti-Ro, anti-La; antiphospholipid antibodies (anti-cardiolipin, anti-beta2glycoprotein1, lupus anticoagulant), ever-low C3; urinary protein/creatinine ratio (uPCR), serum creatinine, albumin, anti-dsDNA (ELISA) and C3 levels – all at the time of biopsy, 12 months afterwards, and at the time of last visit. The same assays were used, over time, for determination of ANA, ENA and anti-dsDNA at UCLH clinical laboratory.

We determined the estimated glomerular filtration rate (eGFR) for all patients using the CKD-EPI creatinine 2009 equation [16].

Renal survival refers to the proportion of patients without ESRD after the diagnosis of LN. ESRD corresponds to the final stage of chronic kidney disease, where renal function is very poor (eGFR < 15) and renal replacement therapy (dialysis or transplant) is necessary.

### Statistical analysis

Statistical analysis was performed with IBM^®^ SPSS^®^ statistics version 22. For categorical variables, we compared groups using Pearson’s chi-squared test. For continuous numerical variables, we used one-way ANOVA, with Tukey’s post-hoc test (Welch’s ANOVA and Games-Howell post-hoc test were used when homogeneity of variances was not met), or Kruskal–Wallis test. A paired-*t* test or a Wilcoxon signed-rank test were performed to compare laboratory results for paired samples, at different time points. Patient and renal cumulative survival were analysed through the Kaplan–Meier method. Patients were censored if they were lost to follow-up or reached the end of the study. Cox regression analysis was performed to investigate predictors of shorter survival: first, each of the possible predictive variables (chosen according to the clinical question) was tested in a univariable Cox regression. For continuous numerical variables showing a significant result, we constructed receiver operating characteristic curves (Supplementary Figs S1 and S2, available at *Rheumatology* online) to investigate whether there was a cut-off for each variable, which could be a good predictor of the event (ESRD or death). Then we tested that cut-off in the Cox regression, to find its hazard ratio. Afterwards, the variables showing significant results were tested together, using a maximum of one covariate for each 10 events in the multivariable Cox regression model. Significance level was defined at 0.05.

## Results

### Patients with MLN have lower anti-dsDNA and higher C3 levels than those with PLN, at the time of biopsy

A total of 187 patients with biopsy-proven PLN (*n* = 135), MLN (*n* = 38) or mixed LN (*n* = 14) were included in this study. However, because of some missing laboratory data, the number of patients for certain analyses was lower (please see detailed number of patients in Table 1). Median time of follow-up was 12 years (interquartile range (IQR) = 13 years, maximum 42 years). Nineteen patients were diagnosed with LN before the age of 16. Age at diagnosis and time between the diagnosis of SLE and LN did not differ between the three groups. However, the groups differ significantly regarding ethnic distribution (Table 1). In fact, there was a significantly higher proportion of MLN amongst Afro-Caribbean patients than amongst Caucasian patients (31% *vs* 15%, *P *= 0.010).

**Table 1:** Comparative description of the UCLH cohort of patients with proliferative, membranous and mixed LN

		Class III and IV	Class V	III+V or IV+V	*P*
Total, *n*		135	38	14	
Females, *n* (%)		123 (91)	33 (87)	11 (79)	0.303
Ethnicity	Caucasian, *n* (%)	**67 (50)**	**12 (32)**	**3 (21)**	**0.037** ^a^
	Afro-Caribbean, *n* (%)	**34 (25)**	**18 (47)**	**6 (43)**	
	Asian, *n* (%)	**34 (25)**	**8 (21)**	**5 (36)**	
Age LN diagnosis (y), mean (S.d.)		29 (12)	29 (14)	26 (8)	0.581
Time SLE-LN (y), median (IQR)		1 (4)	1 (6)	0 (7)	0.842
uPCR at LN diagnosis, median (IQR)		212 (411)	332 (346)	126 (209)	0.370
		*n* = 70	*n* = 26	*n* = 8	
Nephrotic-range proteinuria, *n* (%)		27 (39)	14 (54)	1 (13)	0.098
		*n* = 70	*n* = 26	*n* = 8	
Creatinine at LN diagnosis, median (IQR)		**81 (37)**	**63 (28)**	**70 (55)**	**0.009** ^b^
		*n* = 69	*n* = 25	*n* = 10	
eGFR at LN diagnosis, mean (S.d.)		**95** (**35)**	**120 (34)**	**113 (40)**	**0.007** ^c^
		*n* = 69	*n* = 25	*n* = 10	
Albumin at LN diagnosis, mean (S.d.)		31 (7)	30 (6)	32 (7)	0.647
		*n* = 63	*n* = 21	*n* = 10	
C3 at LN diagnosis, mean (S.d.)		**0.62** (**0.24)**	**0.89** (**0.37)**	**0.66 (0.24)**	**0.018** ^d^
		*n* = 72	*n* = 23	*n* = 9	
Anti-dsDNA at LN diagnosis, median (IQR)		**488 (1541)**	**81 (129)**	**292 (206)**	**0.001** ^e^
		*n* = 72	*n* = 21	*n* = 8	
> 1 renal biopsy, *n* (%)		29 (22)	10 (26)	6 (43)	0.192
Different class in subs. biopsy, *n* (%)		6 (21)	5 (50)	0 (0)	0.058
Ever low C3, *n* (%)		107 (80)	35 (92)	11 (79)	0.203
Ever anti-dsDNA positive, *n* (%)		111 (83)	32 (84)	12 (86)	0.950
Ever anti-Sm positive, *n* (%)		**25 (19)**	**16 (42)**	**6 (43)**	**0.004** ^f^
Ever anti-Ro positive, *n* (%)		54 (40)	16 (42)	10 (71)	0.081
Ever anti-La positive, *n* (%)		21 (16)	3 (8)	4 (29)	0.168
Ever anti-RNP positive, *n* (%)		**42 (31)**	**19 (50)**	**8 (57)**	**0.030** ^g^
Use of antimalarials, *n* (%)		82 (66)	27 (73)	9 (69)	0.732
Use of immunosupressants, *n* (%)		121 (95)	35 (95)	14 (100)	0.669
Use of steroids, *n* (%)		125 (97)	36 (95)	13 (93)	0.668
ESRD – total, *n* (%)		34 (25)	3 (8)	2 (14)	0.056
ESRD – subgroup transplant, *n* (%)		18 (53)	1 (33)	1 (50)	0.126
Deaths, *n* (%)		30 (22)	4 (11)	1 (7)	0.135

Note: There were missing data for some variables, therefore, we present the number of patients analysed for each group below the statistics for those variables. For variables with a statistically significant difference between groups, values are presented in bold.

a Ethnicity: chi-squared test: *P *=0.028 when comparing class III and IV *vs* V; *P *=0.525 for class V *vs* mixed; *P *=0.125 for class III and IV *vs* mixed.

b Creatinine at LN diagnosis: Kruskal–Wallis test was used to compare the three groups; Mann–Whitney’s test was used to compare the groups in pairs: *P *=0.002 when comparing class III and IV *vs* V; *P *=0.270 for class V *vs* mixed; *P *=0.484 for class III and IV *vs* mixed.

c eGFR at LN diagnosis: one-way ANOVA with Tukey HSD post hoc test: *P *=0.008 when comparing class III and IV *vs* V; *P *=0.850 for class V *vs* mixed; *P *=0.289 for class III and IV *vs* mixed.

d C3 at LN diagnosis: Welch ANOVA with Games-Howell post hoc test: *P *=0.009 when comparing class III and IV *vs* V; *P *=0.124 for class V *vs* mixed; *P *=0.903 for class III and IV *vs* mixed.

e Anti-dsDNA at LN diagnosis: Kruskal–Wallis test was used to compare the three groups; Mann–Whitney’s test was used to compare the groups in pairs: *P *<0.001 when comparing class III and IV *vs* V; *P *=0.002 for class V *vs* mixed; *P *=0.558 for class III and IV *vs* mixed.

f Ever anti-Sm positive: chi-squared test: *P *=0.003 when comparing class III and IV *vs* V; *P *=0.961 for class V *vs* mixed; *P *=0.034 for class III and IV *vs* mixed.

g Ever anti-RNP positive: chi-squared test: *P *=0.034 when comparing class III and IV *vs* V; *P *=0.647 for class V *vs* mixed; *P *=0.052 for class III and IV *vs* mixed.

eGFR: estimated glomerular filtration rate, ml/min/1.73 m^2^; ESRD: end-stage renal disease; IQR: interquartile range; LN: lupus nephritis; UCLH: University College London Hospitals; uPCR: urinary protein-creatinine ratio, mg/mmol; y: years. Creatinine is presented in μmol/l, albumin in g/l and C3 in g/l.

At the time of diagnosis of LN, mean eGFR was significantly lower in patients with PLN than MLN or mixed LN (95 *vs* 120 *vs* 113 ml/min/1.73 m^2^ respectively, *P *= 0.007). There was no significant difference between groups regarding levels of uPCR or serum albumin, as all three groups had high levels of proteinuria and serum hypoalbuminemia (Table 1).

At the time of biopsy, mean C3 levels were significantly lower in the PLN (0.62  (S.D. 0.24) g/l) and mixed LN (0.66  (S.D.0.24) g/l) groups than the MLN group (0.89  (S.D. 0.37) g/l) (*P *= 0.018 for difference between groups). In fact, patients with MLN had near normal C3 levels (lower limit of normal at the UCLH laboratory =0.90 g/l). They also had near normal anti-dsDNA levels (median 81 IU/l, IQR 129; upper limit of normal at UCLH =50 IU/l) which were significantly lower than in those with PLN (median 488, IQR 1541) and mixed histology (median 292, IQR 206) (*P *= 0.001 for difference between groups).

Conversely, the groups do not differ in the proportion of patients with ever-positivity for anti-dsDNA, anti-Ro and anti-La antibodies, as well as in the proportion of patients with ever-low C3 levels. However, the proportion of patients ever-positive for anti-Sm and anti-RNP antibodies was lower in patients with PLN. This was not surprising given that this group had a higher proportion of Caucasians, who, in turn, had a significantly lower proportion of anti-Sm (14% *vs* 38% in Afro-Caribbeans and 30% in Asians; *P *= 0.004) and anti-RNP antibodies (21% *vs* 53% in Afro-Caribbeans and 45% in Asians; *P *< 0.001).

The proportion of patients ever treated with antimalarials, steroids or immunosuppressants did not differ between groups. There was also no difference regarding the proportion of patients treated with each immunosuppressant drug: cyclophosphamide (49.5%, 45.9% and 35.7% for PLN, MLN and mixed LN, respectively; *P *= 0.607), mycophenolate mofetil (57%, 59.5% and 78.6%; *P *= 0.297), calcineurin inhibitors (7.8%, 10.8% and 14.3%; *P *= 0.654), azathioprine (66.1%, 70.3% and 78.6%; *P *= 0.605) and B cell-depleting therapy using rituximab (42.2%, 39.5% and 57.1%; *P *= 0.505).

One year after the diagnosis of LN, uPCR decreased significantly and serum albumin increased significantly in patients with both PLN and MLN, but not mixed LN (Table 2). At this time point, levels of C3 increased and levels of anti-dsDNA decreased significantly in patients with PLN and mixed LN, but these changes were not significant in patients with MLN (who had near-normal levels to start with).

**Table 2:** Evolution of laboratory parameters for each group of LN patients

	LN diagnosis	12 months after diagnosis	*P* (diagnosis *vs* 12 M)	7 years after diagnosis^a^	Last visit^a^	*P* (diagnosis *vs* LV)
**Class III and IV**	***n* = 70**	***n* = 69**		***n* = 70**	***n* = 74**	
uPCR, median (IQR)	212 (411)	42 (116)	**<0.001**		21 (39)	0.242
Creatinine, median (IQR)	81 (37)	75 (30)	**0.016**	71 (27)	72 (34)	0.312
eGFR, mean (S.d.)	95 (35)	98 (33)	0.124	96 (30)	87 (33)	0.081
Albumin, mean (S.d.)	31 (7)	39 (6)	**<0.001**		42 (5)	**<0.001**
C3, mean (S.d.)	0.62 (0.24)	0.85 (0.29)	**<0.001**		0.99 (0.22)	**<0.001**
Anti-dsDNA, median (IQR)	488 (1541)	91 (299)	**<0.001**		17 (80)	**<0.001**
**Class V**	***n* = 26**	***n* = 20**		***n* = 24**	***n* = 31**	
uPCR, median (IQR)	332 (346)	39 (76)	**<0.001**		13 (82)	0.266
Creatinine, median (IQR)	63 (28)	67 (29)	0.559	70 (24)	65 (30)	0.523
eGFR, mean (S.d.)	120 (34)	115 (28)	0.559	108 (30)	97 (26)	**0.001**
Albumin, mean (S.d.)	30 (6)	38 (5)	**0.001**		40 (5)	**<0.001**
C3, mean (S.d.)	0.89 (0.37)	0.92 (0.35)	0.708		1.02 (0.26)	0.189
Anti-dsDNA, median (IQR)	81 (129)	34 (79)	0.103		27 (109)	0.616
**Class III+V or IV+V**	***n* = 8**	***n* = 7**		***n* = 6**	***n* = 9**	
uPCR, median (IQR)	126 (209)	148 (262)	0.893		79 (154)	0.686
Creatinine, median (IQR)	70 (55)	69 (21)	0.889	65 (13)	70 (11)	0.767
eGFR, mean (S.d.)	131 (38)	117 (29)	0.683	112 **(**21)	104 (23)	0.620
Albumin, mean (S.d.)	32 (7)	35 (9)	0.362		35 (14)	0.574
C3, mean (S.d.)	0.66 (0.24)	0.78 (0.27)	**0.020**		0.86 (0.32)	0.089
Anti-dsDNA, median (IQR)	292 (206)	136 (155)	**0.046**		204 (368)	0.161

Note: There were missing data for these variables, therefore, the number of patients analysed for each group does not correspond to the total number of patients. For variables with a statistically significant difference between time points, p values are presented in bold.

a For creatinine levels and eGFR at 7 years and at the last visit, patients with ESRD were excluded from this analysis, as they constituted significant outliers.

12 M: 12 months after diagnosis of LN; eGFR: estimated glomerular filtration rate; IQR: interquartile range; LN: lupus nephritis; LV: last visit; uPCR: urinary protein-creatinine ratio.

### Progression to ESRD is faster in patients with PLN and is associated with Afro-Caribbean ethnicity, not taking antimalarials, and eGFR and uPCR at diagnosis and at one year

During the time of follow-up, 39 patients progressed to ESRD and 20 of these had a renal transplant (Table 1). Cumulative renal survival rates at five, 10, 15 and 20 years were 91, 81, 75 and 66%, respectively, for proliferative LN, and 100, 97, 92 and 84% for membranous LN, with a significant difference between these two groups (*P *= 0.028).

Having uPCR above 42 mg/mmol or eGFR below 76 ml/min/1.73 m^2^, one year after the diagnosis of LN, were the strongest predictors of progression to ESRD, in univariable analysis, with hazard ratios (HR) of eight-fold and five-fold, respectively (Table 3). HR for uPCR and eGFR at the time of diagnosis were considerably smaller (2.5 and 2.8 respectively). Other factors associated with increased risk of ESRD were Afro-Caribbean ethnicity (HR = 3.9), PLN (HR = 3.4), not having taken antimalarials (HR = 2.1) and poorly controlled diastolic blood pressure. The effect of uPCR and eGFR at one year remained significant after adjusting for ethnicity, histological class, uPCR and eGFR at the time of diagnosis, the use of antimalarials and diastolic blood pressure (Table 4). eGFR at the time of diagnosis and one year afterwards are strongly correlated (r = 0.778, *P *< 0.001).

**Table 3:** Hazard ratios (HR) for possible predictors of ESRD and mortality, identified by univariable regression Cox analysis

Univariable Cox Regression	HR [95% CI]	*P*
**Predictors of ESRD**		
uPCR ≥ 42 at 12 M	8.081 [1.856, 35.179]	0.005
eGFR ≤ 76 at 12 M	4.985 [1.964, 12.651]	0.001
Ethnicity (AC)	3.861 [1.817, 8.206]	<0.001
Histological class (III or IV)	3.423 [1.049, 11.173]	0.041
eGFR ≤ 82 at LN diagnosis	2.833 [1.156, 6.945]	0.023
uPCR ≥ 262 at LN diagnosis	2.508 [1.062, 5.922]	0.036
No antimalarials	2.180 [1.089, 4.363]	0.028
% Diastolic BP > 80 mmHg	1.016 [1.001, 1.030]	0.032
**Predictors of mortality**		
eGFR ≤ 77 at 12 M	6.591 [2.252, 19.294]	0.001
No antimalarials	3.799 [1.820, 7.929]	<0.001
No steroids	3.719 [1.119, 12.359]	0.032
ESRD	3.299 [1.694, 6.424]	<0.001
UPCR ≥ 67 at 12 M	3.102 [1.039, 9.266]	0.043
Age LN diagnosis > 33	2.278 [1.162, 4.465]	0.016
Ethnicity (AC)	2.241 [1.053, 4.770]	0.036
Year LN diagnosis	0.959 [0.924, 0.996]	0.032
% Diastolic BP > 80 mmHg	1.022 [1.005, 1.039]	0.009

See Supplementary Tables S1 and S2, available at *Rheumatology* online, for full list of variables tested and respective HR.

BP: blood pressure; eGFR: estimated glomerular filtration rate, ml/min/1.73 m^2^; uPCR: urinary protein-creatinine ratio, mg/mmol.

**Table 4:** HR for predictors of ESRD and mortality (variables analysed in pairs with Cox regression)

Cox regression	HR [95% CI]	*P*
Predictors of ESRD		
**uPCR ≥ 42 at 12 M**	**6.365 [1.431**, **28.321]**	**0.015**
**eGFR ≤ 76 at 12 M**	**8.041 [2.831**, **22.834]**	**<0.001**
**uPCR ≥ 42 at 12 M**	**8.027 [1.820**, **35.401]**	**0.006**
Ethnicity (AC)	1.200 [0.361, 3.989]	0.766
**eGFR ≤ 76 at 12 M**	**6.800 [2.490**, **18.572]**	**<0.001**
**Ethnicity (AC)**	**4.138 [1.235**, **13.870]**	**0.021**
**uPCR ≥ 42 at 12 M**	**9.229 [2.104**, **40.481]**	**0.003**
Histological class (III or IV)	286141.61 [<0.001-7.149E+187]	0.953
**eGFR ≤ 76 at 12 M**	**4.300 [1.633**, **11.321]**	**0.003**
Histological class (III or IV)	233853.34 [<0.001-1.219E+192]	0.955
**uPCR ≥ 42 at 12 M**	**5.204 [1.169**, **23.163]**	**0.030**
**eGFR ≤ 82 at LN diagnosis**	**4.660 [1.476**, **14.713]**	**0.009**
**uPCR ≥ 42 at 12 M**	**5.489 [1.226**, **24.574]**	**0.026**
uPCR ≥ 262 at LN diagnosis	2.739 [0.939, 7.992]	0.065
**eGFR ≤ 76 at 12 M**	**4.030 [1.548**, **10.495]**	**0.004**
uPCR ≥ 262 at LN diagnosis	2.195 [0.826, 5.836]	0.115
**uPCR ≥ 42 at 12 M**	**7.119 [1.615**, **31.385]**	**0.010**
**No antimalarials**	**3.828 [1.360**, **10.778]**	**0.011**
**eGFR ≤ 76 at 12 M**	**3.659 [1.337**, **10.011]**	**0.012**
No antimalarials	2.382 [0.857, 6.621]	0.096
**uPCR ≥ 42 at 12 M**	**5.051 [1.097**, **23.349]**	**0.038**
% Diastolic BP > 80 mmHg	1.010 [0.0990, 1.030]	0.347
**eGFR ≤ 76 at 12 M**	**3.554 [1.214**, **10.407]**	**0.021**
% Diastolic BP > 80 mmHg	1.012 [0.992, 1.032]	0.239
Predictors of mortality		
**eGFR ≤ 77 at 12 M**	**5.288 [1.603**, **17.447]**	**0.006**
No antimalarials	1.514 [0476, 4.813]	0.482
**eGFR ≤ 77 at 12 M**	**6.040 [1.995**, **18.283]**	**0.001**
No steroids	2.420 [0.525, 11.159]	0.257
**eGFR ≤ 77 at 12 M**	**3.543 [1.120**, **11.210]**	**0.031**
**ESRD**	**5.807 [1.817**, **18.555]**	**0.003**
**eGFR ≤ 77 at 12 M**	**9.242 [2.418**, **35.319]**	**0.001**
UPCR ≥ 67 at 12 M	2.119 [0.611, 7.354]	0.237
**eGFR ≤ 77 at 12 M**	**14.927 [4.053**, **54.982]**	**<0.001**
**Ethnicity**	**11.506 [2.669**, **49.598]**	**0.001**
**eGFR ≤ 77 at 12 M**	**5.440 [1.770**, **16.719]**	**0.003**
Age LN diagnosis > 33	1.887 [0.638, 5.584]	0.251
**eGFR ≤ 77 at 12 M**	**6.866 [2.290**, **20.581]**	**0.001**
Year LN diagnosis	1.014 [0.938, 1.096]	0.723
**eGFR ≤ 77 at 12 M**	**4.665 [1.308**, **16.641]**	**0.018**
% Diastolic BP > 80 mmHg	1.013 [0.989, 1.037]	0.288

Note: Due to small number of events (ESRD and death), multivariable Cox regression analysis was limited to two covariates. Therefore, instead of including all the eight or nine explicative variables at the same time in a regression model predicting ESRD or death, respectively, we included only a pair of variables at a time. Each possible explicative variable was tested against the variables with the highest HR on univariable analysis (uPCR and eGFR at one year in the case of ESRD, and eGFR at one year in the case of mortality). Variables that keep statistical significance in the multivariable model are presented in bold.

eGFR: estimated glomerular filtration rate, ml/min/1.73 m^2^; uPCR: urinary protein-creatinine ratio, mg/mmol.

**Fig. 1:**
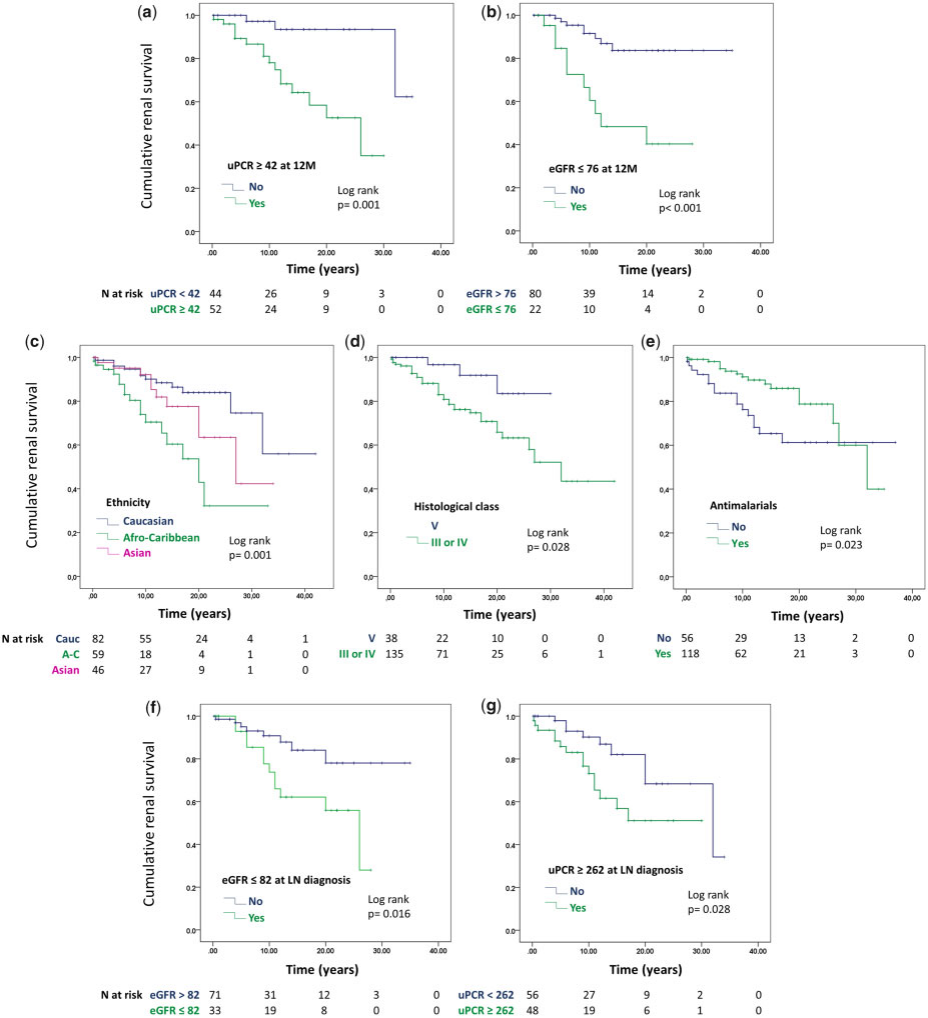
Kaplan–Meier curves showing cumulative renal survival for different groups of patients with membranous and proliferative LN

Fig. 1 shows Kaplan–Meier curves demonstrating the effect of each of the factors shown in Table 3 on development of ESRD over time. All the graphs show significant separation of the curves. With respect to the main aim of this paper, Fig. 1d shows that patients with MLN had significantly better renal survival than those with PLN, although this significance was lost in the multivariable analysis due to the much stronger predictive effect of uPCR and eGFR (Table 4).

At the last follow-up visit, excluding the patients who developed ESRD, there were three patients (8%) with MLN and 18 patients (13%) with PLN with an eGFR below 60 ml/min/1.73 m^2^.

### Mortality does not differ between patients with MLN and PLN but is associated with Afro-Caribbean ethnicity, ESRD and eGFR at one year

Thirty-five of the 187 patients died. The main causes of death were infection (*n* = 11) and cardiovascular (*n* = 11), followed by cancer (*n* = 7) and other causes (*n* = 5). For one patient, the cause of death was uncertain. Cumulative survival figures at five, 10, 15 and 20 years are 94, 86, 80 and 76%, respectively.

**Fig. 2:**
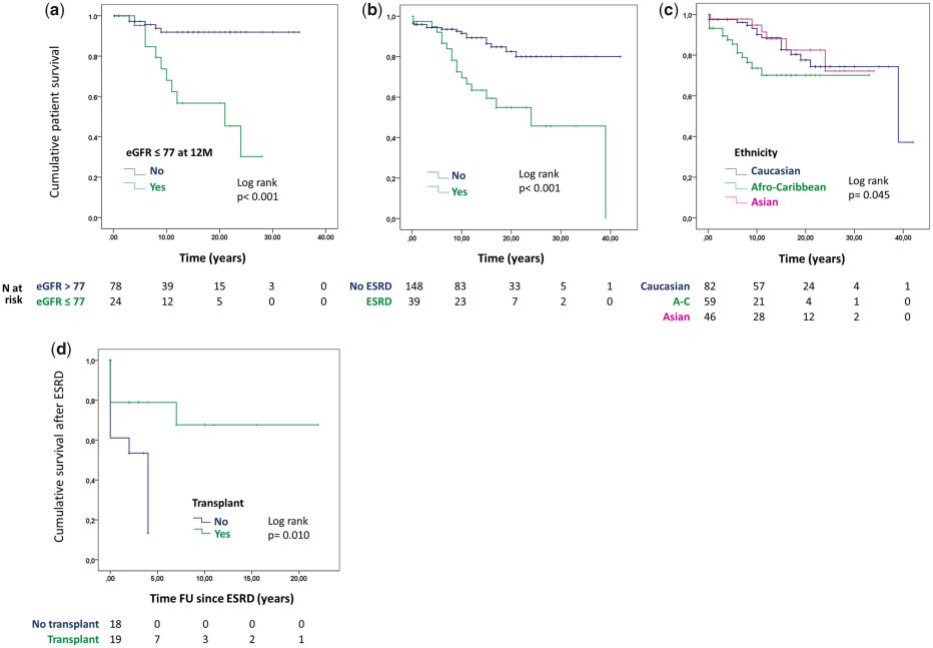
Kaplan–Meier curves showing cumulative patient survival for different groups of patients with MLN and PLN

Table 3 shows that a number of variables were associated with increased mortality on univariable analysis, with eGFR below 77 ml/min/1.73 m^2^, one year after the diagnosis of LN, being associated with the highest HR (6.6) for death. After adjusting for eGFR at one year, only Afro-Caribbean ethnicity and ESRD were independently associated with mortality (Table 4). Fig. 2a–c represents the Kaplan–Meier survival curves showing the significant effects of these three factors. Of note, there was no significant difference in survival between patients with MLN and PLN on Kaplan–Meier analysis (log rank *P *= 0.122). Amongst patients with ESRD, there was a significant difference in survival when comparing those who received a transplant with those who stayed on dialysis (Fig. 2d).

## Discussion

Our results suggest that patients with MLN differ significantly from patients with PLN regarding ethnic background, serologic profiles (anti-dsDNA and C3 levels at the time of diagnosis) and renal survival. We identified uPCR above 42 mg/mmol and eGFR below 76 ml/min/1.73 m^2^, one year after the diagnosis of LN, as the strongest predictors of progression to ESRD. An eGFR below 77 ml/min/1.73 m^2^ one year after the diagnosis of LN, development of ESRD and Afro-Caribbean ethnicity are associated with higher mortality.

Active SLE is often characterized by low complement and raised anti-dsDNA levels, and there were striking differences in these parameters between the membranous and proliferative groups. Other authors have described the differences we found with respect to anti-dsDNA and complement levels at the time of diagnosis. In fact, it is generally accepted that patients with pure MLN can present with normal complement levels and negative anti-dsDNA binding [17, 18]. However, this is the first study directly comparing the actual levels of these serological markers in a large multi-ethnic cohort of patients with MLN or PLN.

The higher proportion of positive anti-Sm and anti-RNP antibodies in patients with MLN and mixed LN probably reflects the higher proportion of Afro-Caribbeans in these two groups. Other studies have shown these antibodies to be more prevalent in Afro-Caribbean patients [19]. It is accepted that the overall prevalence of LN is higher in people of African ancestry, Hispanics and Asians, compared with Caucasians [20]. An epidemiologic study published in 2006, aiming to investigate the prevalence and incidence of biopsy-proven LN in the north-west of England, estimated that the proportion of SLE patients with LN was 10% in Caucasian patients, 27% in Indo-Asian, and 58% in Afro-Caribbean patients [21]. However, no previous studies have shown a difference in ethnic background between patients with PLN and MLN.

Renal survival and mortality rates in our patients are similar to most other cohorts [22], as are the main causes of death [23–26]. In contrast, a Japanese study with 186 LN patients showed lower ESRD and mortality rates; however, more than one-third of the patients included had mesangial LN (class I and II), which is associated with a significantly better prognosis and does not require the same treatment as class III, IV or V [27].

It is important to note that five of our 38 patients classified as MLN on the first renal biopsy developed proliferative changes on a subsequent biopsy (three patients had class IV and two patients had mixed LN), carried out due to relapse of proteinuria. This included two of the only three MLN patients who developed ESRD. Therefore, whereas we are confident that the prognostic predictors for ESRD shown in Fig. 1 are true for PLN, we do not have enough data to confirm their validity for pure MLN. However, a recent retrospective multicentre study by Silva-Fernandez *et al.* analysed the outcome of 150 patients with pure MLN from Spain and the US (65% Caucasians), with a mean follow up of 7.6 years [8]. By the end of follow-up, eight patients (5.3%) had developed ESRD and nine patients (6%) had died. Predictors of ESRD and death were only investigated with univariable logistic regression analysis due to the small number of events. As in our study, the authors found high proteinuria, high serum creatinine and low creatinine clearance at time of diagnosis to be associated with ESRD. Other parameters associated with ESRD were male sex, hypertension and dyslipidaemia. Male sex was also identified in some other studies as a predictor of worse prognosis [28–30]. In our study, which included 20 males, the Cox regression analysis did not show a significant effect of sex on renal or patient survival (Supplementary Tables S1 and S2, available at *Rheumatology* online).

Predictors of death in the study by Silva-Fernandez *et al.* [8] included age, haemodialysis and not having received mycophenolate mofetil or antimalarials. We found both age at diagnosis and the absence of treatment with antimalarials to be associated with death, on univariable analysis. However, the effect was not significant after adjusting for eGFR. Several other studies mention the protective role of antimalarials in patients with LN [8, 31]. We did not analyse the effect of each individual immunosuppressant separately, but did not find a significant effect from the use of immunosuppressants as a whole. Our patients on dialysis also had poorer survival than those who received a transplant, and this has been already shown by other studies [32, 33]. ESRD in general has been found to predict higher mortality in patients with LN [34].

Although most studies suggest baseline proteinuria and serum creatinine as predictors of prognosis [8, 28–31], several other studies, like ours, support the importance of renal parameters at 12 months [6, 35, 36]. Dall’Era *et al.* looking at long-term outcomes in 76 patients of the Euro-Lupus Nephritis Cohort, found proteinuria below 0.8 g/day, 12 months after beginning treatment, to be the single best predictor of good long-term renal function (sensitivity 81% and specificity 78%) [6]. Similarly, in 90 patients from the MAINTAIN Nephritis Trial, a cut-off of 0.7 g/day for proteinuria was the best predictor of renal outcome (sensitivity 71%, specificity 75%) [36]. Furthermore, a Japanese study with 81 patients followed for a median period of 4.25 years showed that achieving a complete renal remission (uPCR < 50 mg/mmol and a normal or near normal eGFR) at 12 months after induction therapy was associated with a higher flare-free rate [35].

Different studies showed that sustained renal remission is associated with a better prognosis in LN [37], notably with reduced progression to chronic kidney disease, ESRD and mortality [38]. A Korean study suggested that, in diffuse PLN, remission of proteinuria is an independent prognostic marker associated with improved renal and patient survival, regardless of the time between the biopsy and the normalization of proteinuria or the recurrence of proteinuria after remission [7].

Alarcón and collaborators, in the USA, showed that while outcomes were poorer in ethnic minority groups, poverty, rather than ethnicity, was independently associated with mortality of SLE patients [20, 39]. In the UK, where access to the health system is free, socioeconomic factors should play a less preponderant role, as suggested by a 25-year follow up of our cohort [40]. A further formal study, assessing socioeconomic factors such as education, employment status and others, would help to answer this question definitively.

One of the main limitations of our study is the high number (44%) of patients with some missing laboratory data at the time of the renal biopsy. These patients could not be included in the Cox regression analyses investigating the effect of those specific laboratory parameters on ESRD and death. However, patients included in the analyses do not differ significantly from those with missing data regarding sex, age of diagnosis, class of LN, treatment received, ESRD and deaths (Supplementary Table S3, available at *Rheumatology* online).

We believe that the main strengths of our study relate to our large multi-ethnic cohort with a very long period of follow-up, often exceeding 30 years. We used survival analysis to investigate predictors of ESRD and death, which, as opposed to linear or logistic regression, takes into account the time when the event occurred rather than only whether it occurred or not. Furthermore, this kind of analysis considers the subjects in whom the event did not occur during the time of follow-up (censored); therefore, the risk of bias due to underestimating the occurrence of the event is lower.

In conclusion, patients with MLN and PLN differ significantly regarding serological profiles and renal survival, suggesting different pathogenesis. Notably, anti-dsDNA antibodies, which have been associated with the pathogenesis of PLN, may not have a preponderant role in MLN. Antigen discovery studies using proteomic techniques, for instance, would be important to investigate the antigens and antibodies involved in LN. Studies with other multi-ethnic cohorts would be important to confirm our finding of a higher proportion of MLN amongst patients with African ancestry. This might represent a different genetic susceptibility associated with MLN. Proteinuria and renal function at year one appear to be the best predictors of progression to ESRD. Renal function at year one, ESRD and ethnicity are associated with mortality.

## Supplementary Material

keaa103_supplementary_data
