# Supplementary material for: Outcomes of membranous and proliferative lupus nephritis – analysis of a single-centre cohort with more than 30 years of follow-up
Source: Rheumatology (Oxford). 2020 Apr 17;59(11):3314–23. doi: 10.1093/rheumatology/keaa103 (PMC7590413; doi:10.1093/rheumatology/keaa103)
Supplement: keaa103_supplementary_data [file keaa103_supplementary_data.zip › rhe-19-1984-File004.docx]

**SUPPLEMENTARY MATERIAL**

**Supplementary table S1: Hazard ratios for possible predictors of ESRD, identified by univariable COX regression analysis.**

| Univariable COX Regression | **HR [95%CI]** | **p** |
| --- | --- | --- |
| **Creatinine ≥ 77 at 12M** | **6.901 [1.996-23.851]** | **0.002** |
| **Creatinine ≥ 82 at LN diagnosis** | **5.325 [1.773-15.995]** | **0.002** |
| **uPCR ≥ 42 at 12M** | **8.081 [1.856-35.179]** | **0.005** |
| **uPCR ≥ 67 at 12M** | **5.031 [1.772-14.285]** | **0.002** |
| **eGFR ≤ 76 at 12M** | **4.985 [1.964-12.651]** | **0.001** |
| **Ethnicity (AC)** | **3.861 [1.817-8.206]** | **0.000** |
| **Histological class (III or IV)** | **3.423 [1.049-11.173]** | **0.041** |
| **eGFR ≤ 82 at LN diagnosis** | **2.833 [1.156-6.945]** | **0.023** |
| **uPCR ≥ 262 at LN diagnosis** | **2.508 [1.062-5.922]** | **0.036** |
| **No antimalarials** | **2.180 [1.089-4.363]** | **0.028** |
| **Creatinine at 12M** | **1.024 [1.015-1.033]** | **0.000** |
| **Creatinine at LN diagnosis** | **1.016 [1.006-1.026]** | **0.002** |
| **uPCR at 12M** | **1.004 [1.002-1.006]** | **0.000** |
| **uPCR at LN diagnosis** | **1.001 [1.000-1.002]** | **0.006** |
| **Albumin at 12M** | **0.924 [0.856-0.996]** | **0.040** |
| **eGFR at 12M** | **0.968 [0.952-0.985]** | **0.000** |
| **eGFR at LN diagnosis** | **0.981 [0.967-0.996]** | **0.012** |
| **% Diastolic BP > 80mmHg ^1^** | **1.016 [1.001-1.030]** | **0.032** |
| Albumin at LN diagnosis | 0.945 [0.873-1.124] | 0.167 |
| C3 at LN diagnosis | 0.290 [0.043-1.951] | 0.203 |
| Anti-dsDNA at LN diagnosis | 1.000 [0.999-1.001] | 0.296 |
| No steroids | 2.263 [0.537-9.538] | 0.266 |
| No immunosupressants | 1.123 [0.263-4.802] | 0.875 |
| Age LN diagnosis | 0.997 [0.970-1.025] | 0.823 |
| Year LN diagnosis | 0.999 [0.960-0.041] | 0.978 |
| Male sex | 0.955 [0.338-2.693] | 0.930 |
| Diabetes | 1.126 [0.269-4.712] | 0.871 |
| Antiphospholipid syndrome | 1.166 [0.454-2.995] | 0.749 |
| Anti-Sm | 1.107 [0.503-2.436] | 0.801 |
| Anti-RNP | 1.108 [0.559-2.193] | 0.769 |
| % Systolic BP>130mmHg **^1^** | 1.007 [0.993 - 1.021] | 0.335 |

^1^These figures represent the percentage of clinic visits at which the patients’ blood pressure was recorded as systolic > 130mmHg or diastolic > 80mmHg and were included in the analysis to assess the effect of uncontrolled hypertension.

**Supplementary table S2: Possible predictors of death, identified by univariable COX Regression analysis.**

| Univariable COX Regression | **HR [95%CI]** | **p** |
| --- | --- | --- |
| **eGFR ≤ 77 at 12M** | **6.591 [2.252 - 19.294]** | **0.001** |
| **Creatinine ≥ 77 at 12M** | **5.226 [1.473 - 18.542]** | **0.010** |
| **No antimalarials** | **3.799 [1.820 - 7.929]** | **0.000** |
| **No steroids** | **3.719 [1.119 - 12.359]** | **0.032** |
| **ESRD** | **3.299 [1.694 - 6.424]** | **0.000** |
| **UPCR ≥ 67 at 12M** | **3.102 [1.039 - 9.266]** | **0.043** |
| **Ethnicity (AC)** | **2.241 [1.053 - 4.770]** | **0.036** |
| **Age LN diagnosis > 33** | **2.278 [1.162 - 4.465]** | **0.016** |
| **Age LN diagnosis** | **1.033 [1.009 - 1.058]** | **0.008** |
| **Creatinine at 12M** | **1.024 [1.014 - 1.033]** | **0.000** |
| **% Diastolic BP>80mmHg ^1^** | **1.022 [1.005 -1.039]** | **0.009** |
| **Creatinine at LN diagnosis** | **1.014 [1.002 - 1.025]** | **0.022** |
| **eGFR at 12M** | **0.968 [0.950 - 0.985]** | **0.000** |
| **Year LN diagnosis** | **0.959 [0.924 - 0.996]** | **0.032** |
| Year LN diagnosis ≤ 1998 | 1.915 [0.892 - 5.319] | 0.794 |
| No immunosupressants | 2.710 [0.821 - 8.938] | 0.102 |
| Histological class (III and IV) | 2.217 [0.779 - 6.310] | 0.136 |
| Male sex | 0.520 [0.125 - 2.170] | 0.370 |
| Diabetes | 1.106 [0.241 - 5.071] | 0.897 |
| % Systolic BP>130mmHg ^1^ | 1.009 [0.994 - 1.025] | 0.240 |
| Antiphospholipid syndrome | 1.393 [0.538 - 3.608] | 0.495 |
| Anti-Sm | 0.516 [0.181 - 1.472] | 0.216 |
| Anti-RNP | 2.003 [0.871 - 4.603] | 0.102 |
| Creatinine ≥ 82 at LN diagnosis | 2.129 [0.771 - 5.875] | 0.145 |
| eGFR at LN diagnosis | 0.989 [0.975 - 1.004] | 0.158 |
| uPCR at LN diagnosis | 1.001 [0.999 - 1.002] | 0.286 |
| Albumin at LN diagnosis | 0.991 [0.910 - 1.078] | 0.829 |
| C3 at LN diagnosis | 1.217 [0.279 - 5.319] | 0.794 |
| Anti-dsDNA at LN diagnosis | 1.000 [1.000 - 1.001] | 0.086 |

^1^These figures represent the percentage of clinic visits at which the patients’ blood pressure was recorded as systolic > 130mmHg or diastolic > 80mmHg and were included in the analysis to assess the effect of uncontrolled hypertension.

Supplementary table S3: Comparison between patients with and without missing data regarding laboratory parameters at the time of the renal biopsy.

|  | | **Included cases** | **Missing cases** | **p** |
| --- | --- | --- | --- | --- |
| **Total**, N | | 104 | 83 |  |
| **Females,** N (%) | | 89 (86) | 78 (94) | 0.065 |
| **Ethnicity** | **Caucasian**, N (%) | 40 (39) | 42 (51) | **0.027** |
|  | **Afro-Caribbean**, N (%) | 30 (29) | 28 (34) |  |
|  | **Asian**, N (%) | 34 (33) | 13 (16) |  |
| **Age LN diagnosis**(y), mean ± SD | | 29 ± 12 | 29 ± 12 |  |
| **Histology** | **Proliferative**, N (%) | 70 (67) | 65 (78) | 0.191 |
|  | **Membranous**, N (%) | 26 (25) | 12 (15) |  |
|  | **Mixed**, N (%) | 8 (8) | 6 (7) |  |
| **Use of antimalarials**, N (%) | | 69 (68) | 49 (67) | 0.868 |
| **Use of immunosupressants**, N (%) | | 97 (95) | 73 (95) | 0.929 |
| **Use of steroids**, N (%) | | 97 (94) | 77 (99) | 0.116 |
| **B-cell depletion therapy**, N (%) | | 49 (47) | 31 (37) | 0.180 |
| **ESRD – total, N** (%) | | 24 (23) | 15 (18) | 0.403 |
| **ESRD – subgroup transplant, N** (%) | | 12 (12) | 9 (11) | 0.881 |
| **Deaths, N** (%) | | 21 (20) | 14 (17) | 0.562 |

Supplementary figure legend

**Supplementary figure S1: ROC curves for significant predictors of ESRD on COX regression analysis.

Supplementary figure S2: ROC curves for significant predictors of death on COX regression analysis.**
