# Supplementary figures and images for: Outcomes of membranous and proliferative lupus nephritis – analysis of a single-centre cohort with more than 30 years of follow-up
Source: Rheumatology (Oxford). 2020 Apr 17;59(11):3314–23. doi: 10.1093/rheumatology/keaa103 (PMC7590413; doi:10.1093/rheumatology/keaa103)

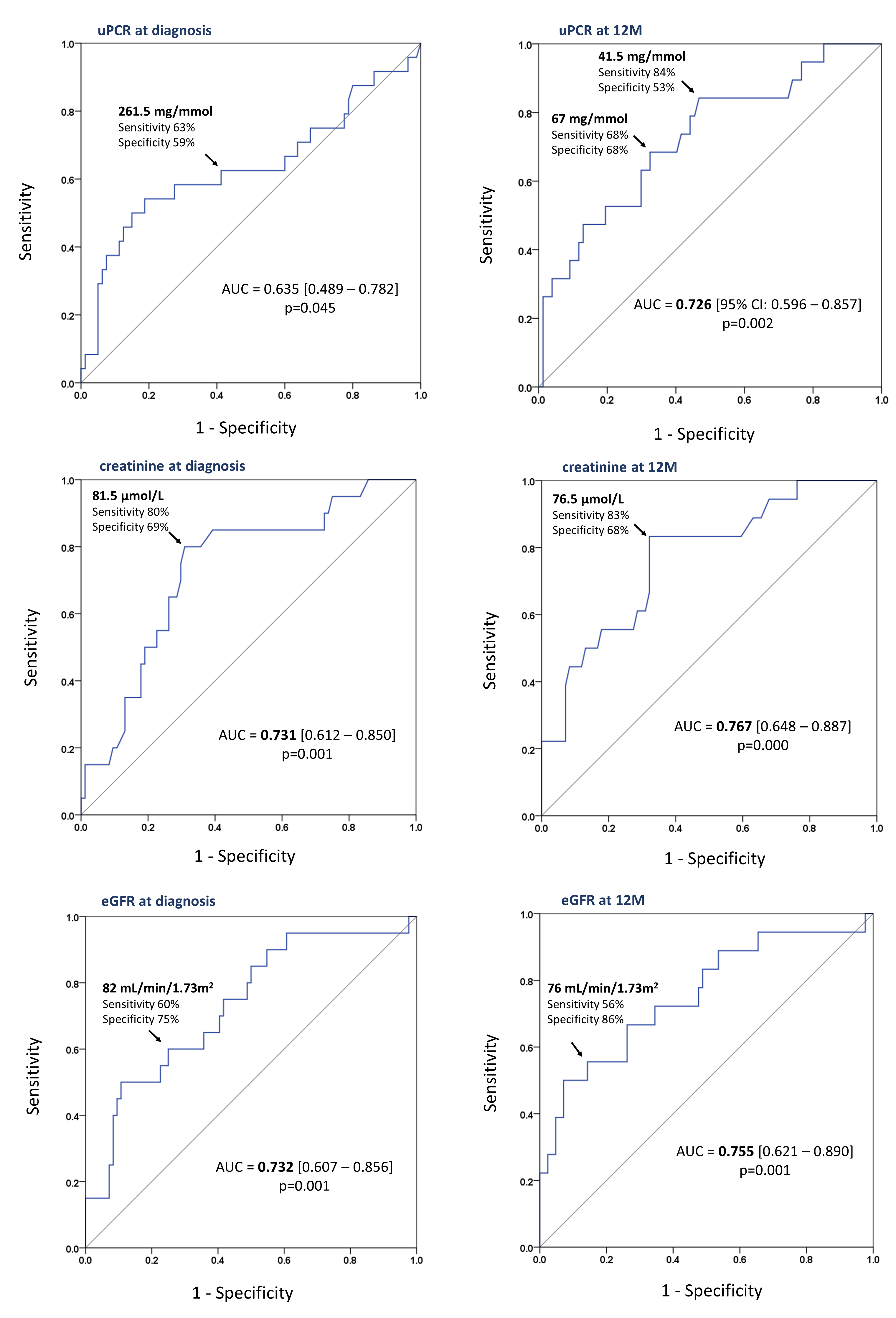

Supplement: keaa103_supplementary_data [file keaa103_supplementary_data.zip › rhe-19-1984-File005.png]

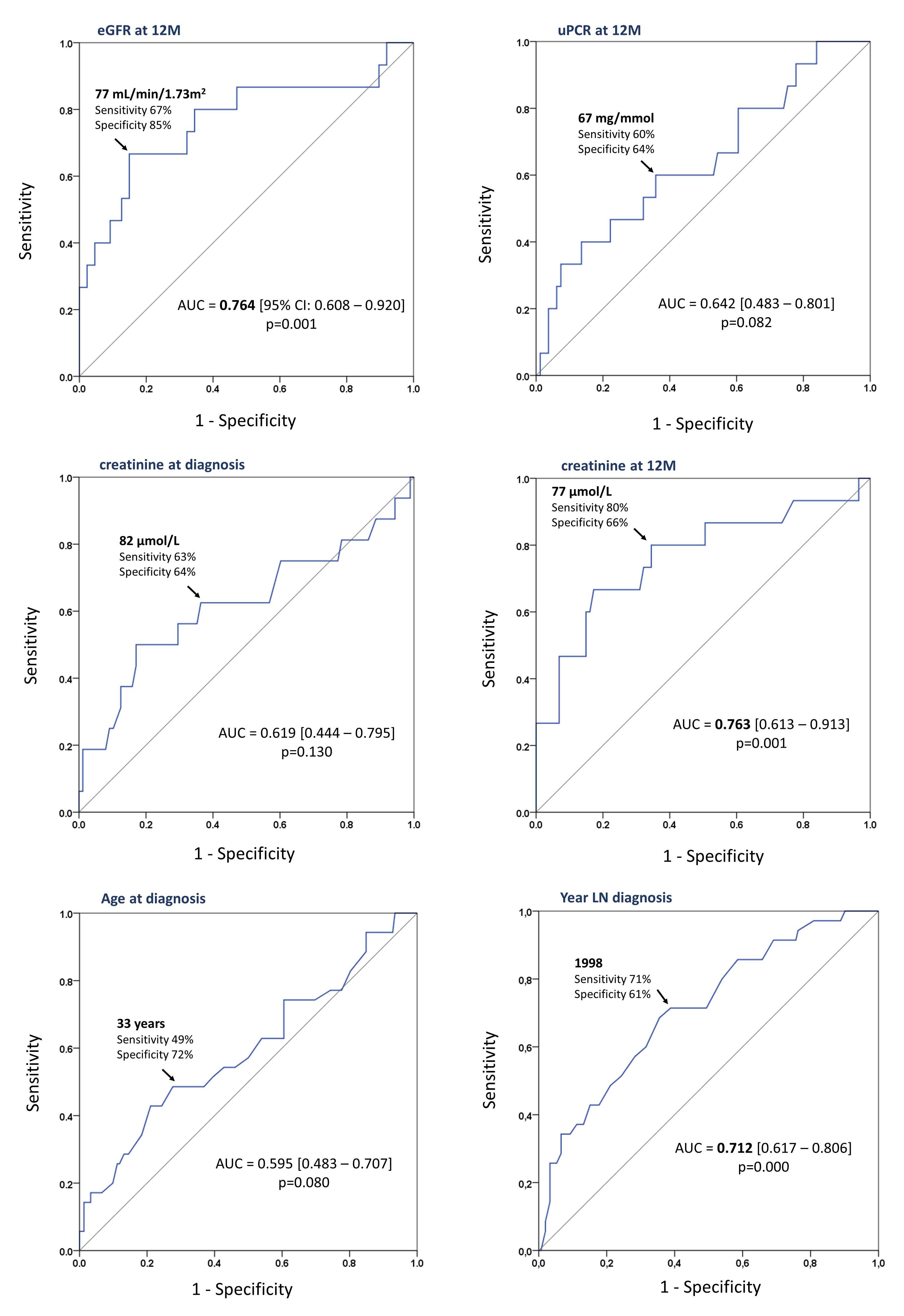

Supplement: keaa103_supplementary_data [file keaa103_supplementary_data.zip › rhe-19-1984-File006.png]
